# MAFB shapes human monocyte–derived macrophage response to SARS-CoV-2 and controls severe COVID-19 biomarker expression

**DOI:** 10.1172/jci.insight.172862

**Published:** 2023-12-22

**Authors:** Miriam Simón-Fuentes, Israel Ríos, Cristina Herrero, Fátima Lasala, Nuria Labiod, Joanna Luczkowiak, Emilia Roy-Vallejo, Sara Fernández de Córdoba-Oñate, Pablo Delgado-Wicke, Matilde Bustos, Elena Fernández-Ruiz, Maria Colmenares, Amaya Puig-Kröger, Rafael Delgado, Miguel A. Vega, Ángel L. Corbí, Ángeles Domínguez-Soto

**Affiliations:** 1 Myeloid Cell Laboratory, Centro de Investigaciones Biológicas, CSIC, Madrid, Spain.; 2 Immunometabolism and Inflammation Unit, Instituto de Investigación Sanitaria Gregorio Marañón (IiSGM), Madrid, Spain.; 3 Instituto de Investigación Hospital Universitario 12 de Octubre (imas12), Universidad Complutense School of Medicine, Madrid, Spain.; 4 Rheumatology Department, University Hospital La Princesa and Research Institute, Madrid, Spain.; 5 Molecular Biology Unit, University Hospital La Princesa and Research Institute, Universidad Autónoma de Madrid, Madrid, Spain.; 6 Institute of Biomedicine of Seville (IBiS), Spanish National Research Council (CSIC), University of Seville, Virgen del Rocio University Hospital (HUVR), Seville, Spain.

**Keywords:** COVID-19, Immunology, Cellular immune response, Macrophages, Molecular pathology

## Abstract

Monocyte-derived macrophages, the major source of pathogenic macrophages in COVID-19, are oppositely instructed by macrophage CSF (M-CSF) or granulocyte macrophage CSF (GM-CSF), which promote the generation of antiinflammatory/immunosuppressive MAFB^+^ (M-MØ) or proinflammatory macrophages (GM-MØ), respectively. The transcriptional profile of prevailing macrophage subsets in severe COVID-19 led us to hypothesize that MAFB shapes the transcriptome of pulmonary macrophages driving severe COVID-19 pathogenesis. We have now assessed the role of MAFB in the response of monocyte-derived macrophages to SARS-CoV-2 through genetic and pharmacological approaches, and we demonstrate that MAFB regulated the expression of the genes that define pulmonary pathogenic macrophages in severe COVID-19. Indeed, SARS-CoV-2 potentiated the expression of MAFB and MAFB-regulated genes in M-MØ and GM-MØ, where MAFB upregulated the expression of profibrotic and neutrophil-attracting factors. Thus, MAFB determines the transcriptome and functions of the monocyte-derived macrophage subsets that underlie pulmonary pathogenesis in severe COVID-19 and controls the expression of potentially useful biomarkers for COVID-19 severity.

## Graphical abstract

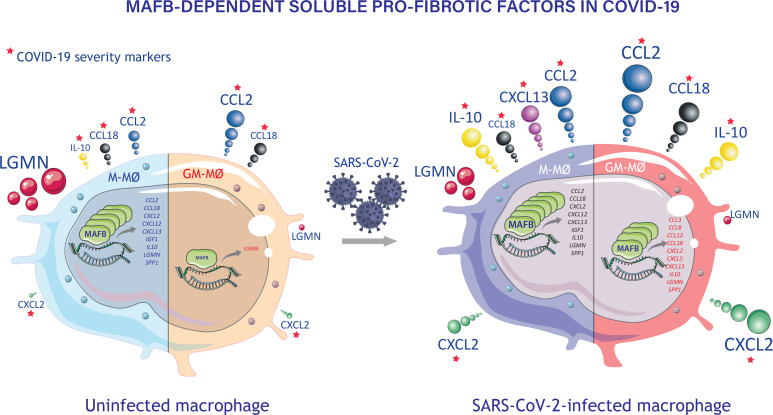

## Introduction

Macrophages display functional heterogeneity and plasticity in homeostasis and during inflammatory responses, when they initially exert proinflammatory activities and later contribute to tissue repair and inflammation resolution (1, 2). The acquisition of macrophage effector functions is largely determined by their ontogeny (fetal origin versus monocyte derived), tissue location, and the prevailing extracellular cytokine millieu (3–5). Regarding ontogeny, monocyte-derived macrophages, which replenish tissue-resident macrophages only in some tissues (e.g., gut) (6), are the major source of pathogenic macrophages in inflamed tissues affected by inflammatory diseases and COVID-19 (7), and they are oppositely instructed by macrophage CSF (M-CSF) or granulocyte macrophage CSF (GM-CSF) (8–14). GM-CSF prompts monocyte-derived macrophages (GM-CSF–dependent monocyte-derived macrophages [GM-MØ]) with enhanced proinflammatory (IL-10^lo^TNF^hi^IL-23^hi^IL-6^hi^) and immunogenic activity, and their transcriptome resembles lung alveolar macrophages (15) and inflammatory macrophages in vivo (12, 16–18). Conversely, M-CSF drives the generation of antiinflammatory, profibrotic, and immunosuppressive (IL-10^hi^TNF^lo^IL-23^lo^IL-6^lo^) monocyte-derived macrophages (M-CSF–dependent monocyte-derived macrophages [M-MØ]), whose transcriptome resembles that of homeostatic tissue-resident and protumoral in vivo macrophages (1, 2, 19).

The homeostatic and reparative transcriptional profile of human M-MØ is shaped by MAF and MAFB (20–22), 2 closely related transcription factors that regulate the differentiation of numerous cell lineages (23) and whose levels and activity is regulated by GSK3β (23). In the mouse hematopoietic lineage, Mafb expression is mostly restricted to tissue-resident macrophages, where it promotes macrophage differentiation (24, 25) and inhibits stemness and self-renewal of monocytes and macrophages in cooperation with Maf (26–28), which itself promotes IL-10 and inhibits IL-12 production (29, 30). Conversely, the production of IL-10 is MAFB dependent in the case of human M-MØ (20, 31), and MAFB directly contributes to the macrophage reprogramming action of methotrexate (32) and LXR ligands (33).

Monocyte-derived macrophages lie in the center of severe COVID-19 pathogenesis (34). Although macrophages exhibit an hyperinflammatory phenotype and are responsible for pathogenesis in severe COVID-19 (34), viral entry, productive infection, and activation by SARS-CoV-2 has been a matter of debate (35–37), and the presence of SARS-CoV-2 RNA in tissue-resident alveolar macrophages from autopsied patients who had COVID-19 (38–41) has been interpreted as either capture of infected bystander cells or active virus replication (35). Macrophages are not permissive to productive SARS-CoV-2 replication in vitro (36, 37), and ACE2-independent macrophage capture of SARS-CoV-2 virus particles by lectins or FcγRs-dependent entry of opsonized virus promotes proinflammatory responses but does not lead to productive viral infection (35, 42–46); furthermore, it triggers inflammatory cell death (pyroptosis mediated by activation of NLRP3 and AIM2 inflammasomes, caspase-1, and gasdermin D). This inflammatory cell death aborts the production of infectious viruses and leads to systemic inflammation (42). However, ACE2 expression, only present on a subset of macrophages at sites of SARS-CoV-2 infection in humans, appears to restore macrophage permissiveness to virus replication and production of infectious progeny virions (35, 43, 47). In fact, data from humanized mouse models indicate that tissue-resident human macrophages are permissive to SARS-CoV-2 infection and that the CD16- and ACE2-dependent viral infection leads to inflammasome activation and pyroptosis, which prevents a productive viral cycle and contributes to lung inflammation (46). Extensive single-cell RNA-Seq (scRNA-Seq) on broncho-alveolar lavage and lungs from affected individuals has revealed a huge increase in proinflammatory and profibrotic monocyte-derived macrophages as well as a considerable reduction of tissue-resident alveolar macrophages (39, 48, 49). Of note, the transcriptome of pathogenic pulmonary monocyte-derived macrophage subsets has been found to resemble that of M-MØ (20, 50), which led us to hypothesize a role for MAFB during macrophage responses to SARS-CoV-2 infection (50). We have now directly assessed the role of MAFB in the response of human monocyte–derived macrophages to SARS-CoV-2 infection by using a combination of transcriptional and functional analysis on SARS-CoV-2–exposed M-MØ and GM-MØ. Our results demonstrate the involvement of MAFB in the expression of the genes that characterize pathogenic macrophage clusters in severe COVID-19 and reveal that MAFB expression is potentiated in infected M-MØ and GM-MØ, where it controls the expression of profibrotic factors (IL-10, CCL2, CCL18, CXCL12, CXCL13, SPP1) and neutrophil-attracting chemokines like CXCL2 and CXCL5, some of which act as potentially useful prognostic biomarkers for COVID-19 severity. As a whole, our results reveal a critical role of MAFB in shaping the transcriptome and functional ability of the monocyte-derived macrophage subsets that underlie the pathogenesis of pulmonary fibrosis in severe COVID-19.

## Results

### The MAFB-dependent transcriptome of M-MØ is significantly overexpressed in pathogenic pulmonary macrophages from patients with severe COVID-19.

**Figure 1:**
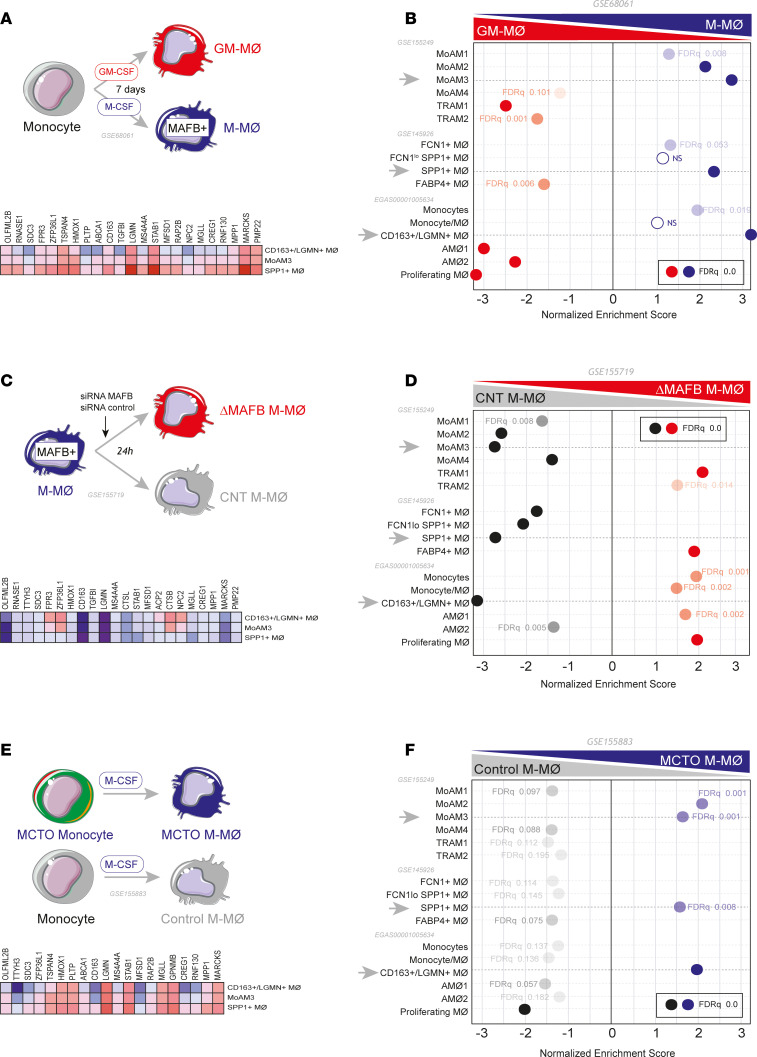
Overexpression of MAFB-dependent genes in the transcriptome of pathogenic pulmonary macrophage subsets in severe COVID-19. (**A**) Schematic representation of the generation of M-MØ and GM-MØ. (**B**) Summary of GSEA of the gene sets that characterize the macrophage subsets identified in severe COVID-19 (39, 48, 49) on the ranked comparison of the transcriptomes of M-MØ versus GM-MØ (GSE68061). Leading edge analysis of the GSEA of the genes that define the MoAM3, SPP1^+^, or CD163^+^/LGMN^+^ subsets on the ranked comparison of the transcriptomes of M-MØ versus GM-MØ is shown under schematic representation. (**C**) Schematic representation of the generation of ΔMAFB M-MØ and control M-MØ (CNT M-MØ) before RNA isolation and RNA-Seq (GSE155719). (**D**) Summary of GSEA of the gene sets that characterize the macrophage subsets identified in severe COVID-19 (39, 48, 49) on the ranked comparison of the transcriptomes of ΔMAFB M-MØ versus CNT M-MØ. Leading edge analysis of the GSEA of the genes that define the MoAM3, SPP1^+^, or CD163^+^/LGMN^+^ subsets on the ranked comparison of the transcriptomes of ΔMAFB M-MØ versus CNT M-MØ is shown under schematic representation. (**E**) Schematic representation of the in vitro generation of M-MØ from a patient with MCTO (MCTO M-MØ) or healthy controls (Control M-MØ) before RNA isolation and RNA-Seq (GSE155883). (**F**) Summary of GSEA of the gene sets that characterize the macrophage subsets identified in severe COVID-19 (39, 48, 49) on the ranked comparison of the transcriptomes of MCTO M-MØ versus Control M-MØ. Leading edge analysis of the GSEA of the genes that define the MoAM3, SPP1^+^, or CD163^+^/LGMN^+^ subsets on the ranked comparison of the transcriptomes of MCTO M-MØ versus Control M-MØ is shown under schematic representation.

We have previously hypothesized that the MAFB-dependent transcriptome of M-MØ (20) is overexpressed in pathogenic pulmonary monocyte-derived macrophages (49) from patients with severe COVID-19 (50). Analysis of more recent transcriptional information on pathogenic lung macrophages from COVID-19 (39, 48) provided further support for this premise. Specifically, the transcriptome of M-MØ (Figure 1A) significantly overexpressed the gene sets that define pathogenic lung macrophage subsets identified as SPP1^+^ MØ (Group 3, GSE145926) (49), MoAM3 (GSE155249) (39), or CD163^+^/LGMN^+^ MØ (EGAS00001005634) (48) clusters in distinct studies (Figure 1B and Supplemental Figure 1A; supplemental material available online with this article; https://doi.org/10.1172/jci.insight.172862DS1). Altogether, these analyses point to a role for MAFB in shaping the transcriptome of pathogenic pulmonary macrophages in severe COVID-19.

To gather additional support for our hypothesis, we next modulated MAFB expression levels in M-MØ through genetic and pharmacological approaches. First, MAFB expression was knocked down in M-MØ using MAFB-specific siRNA (Figure 1C and Supplemental Figure 1, B and C), and the gene profile of the resulting MFAB knockdown (ΔMAFB) M-MØ was determined. Compared with M-MØ transfected with a control siRNA (CNT M-MØ), MAFB knockdown diminished the expression of M-MØ–specific genes (“Antiinflammatory gene set”, GSE68061) (12, 14), including *MAF*, and enhanced the expression of GM-MØ–specific genes (“Proinflammatory gene set”) (12, 14) (Supplemental Figure 1, D–F). More importantly, MAFB knockdown led to a significant downregulation of the gene sets that define the pathogenic SPP1^+^ MØ (GSE145926) (49), MoAM3 (GSE155249) (39), or CD163^+^/LGMN^+^ MØ (accession number EGAS00001005634) (48) subsets in severe COVID-19 (Figure 1D and Supplemental Figure 1A). In fact, the genes that define the transcriptome of the profibrotic CD163^+^/LGMN^+^ MØ subset included a large number of MAFB-dependent genes (47%, 111 out of 237), and similar enrichments were seen in MoAM3 and SPP1^+^ MØ subsets (data not shown). Next, we determined the gene signature of M-MØ from a patient with multicentric carpotarsal osteolysis (MCTO, MCTO M-MØ) (GSE155883) (20) (Figure 1E and Supplemental Figure 1G), a pathology caused by mutations that enhance MAFB protein stability and expression (51) (Supplemental Figure 1H). MCTO M-MØ not only showed a positive enrichment of MAFB-dependent genes (Supplemental Figure 1I) but exhibited a high overrepresentation of the gene sets that define the profibrotic MoAM3 (39), SPP1^+^ (49), and CD163^+^/LGMN^+^ (48) macrophage subsets from lungs of patients with severe COVID-19 (Figure 1F). Moreover, comparison of the leading edge of the distinct GSEA of MoAM3, SPP1^+^, and CD163^+^/LGMN^+^ subsets revealed the common presence of genes like *LGMN*, *CD163*, *HMOX1*, and *STAB1*, which define these macrophage subsets and are associated to fibrotic processes (Supplemental Figure 1J). Altogether, analyses of monocyte-derived macrophages with altered MAFB expression (ΔMAFB M-MØ and MCTO M-MØ) fully support a role for MAFB in shaping the transcriptome of the pathogenic macrophage subsets in severe COVID-19.

### GSK3β inhibition prompts the acquisition of the transcriptional profile of severe COVID-19 pathogenic pulmonary macrophages via MAFB.

**Figure 2:**
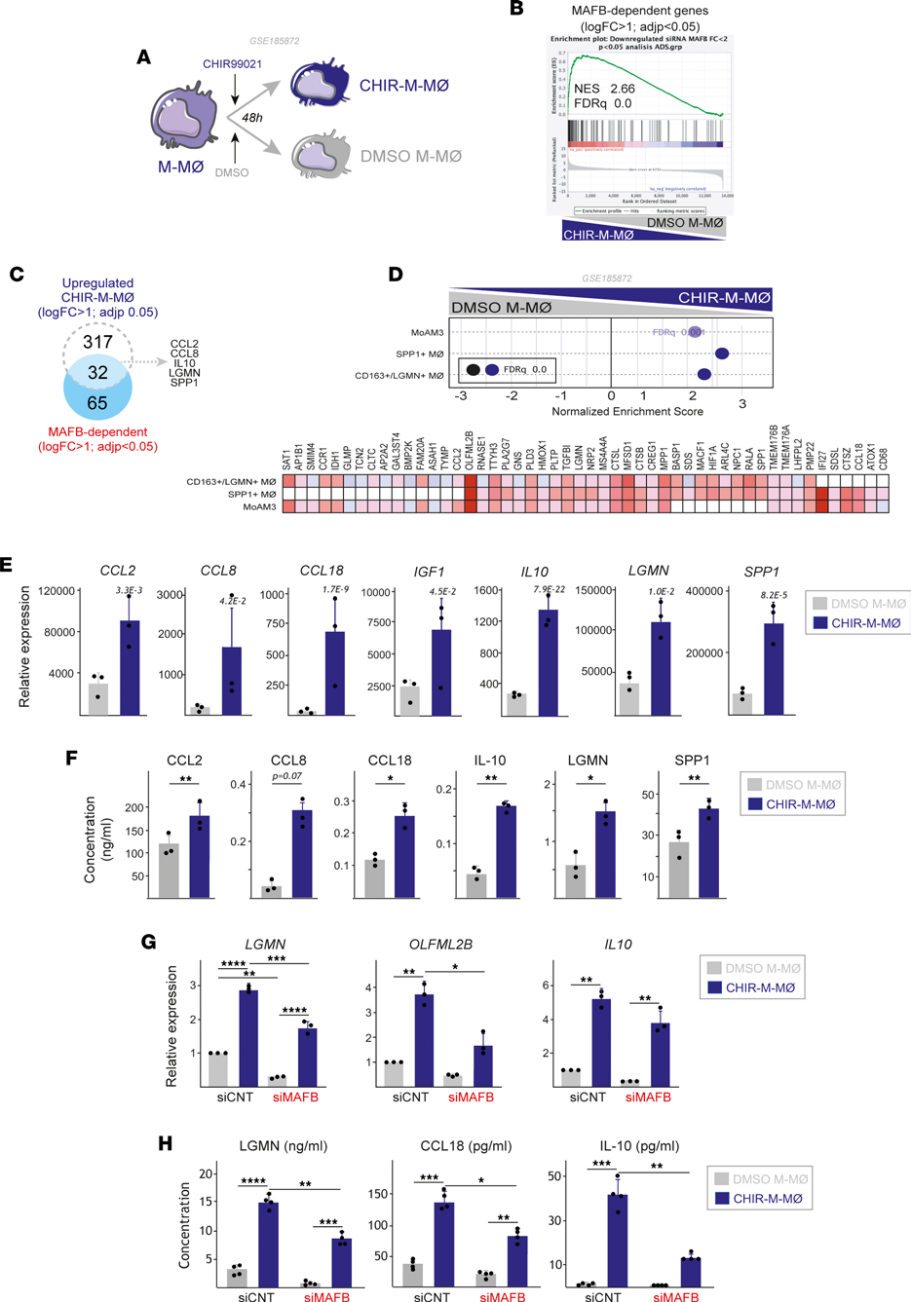
GSK3β inhibition upregulates MAFB-dependent genes and the expression of the gene sets that define pathogenic macrophage subsets in severe COVID-19. (**A**) Schematic representation of the treatment of M-MØ to CHIR99021 (10 μM, CHIR-M-MØ) or DMSO (DMSO M-MØ). (**B**) GSEA of the MAFB-dependent gene set on the comparison of CHIR-M-MØ and DMSO M-MØ transcriptomes. (**C**) Overlap between the genes upregulated (|log2FC| > 1; *P*_adj_ < 0.05) in CHIR-M-MØ (relative to DMSO M-MØ) and MAFB-dependent genes. (**D**) GSEA summary of gene sets characterizing macrophage subsets identified in severe COVID-19 (39, 48, 49) on the comparison of CHIR-M-MØ and DMSO M-MØ transcriptomes. The source of the original data is indicated. Leading edge analysis of the GSEA of the genes that define the MoAM3, SPP1^+^, or CD163^+^/LGMN^+^ subsets on the ranked comparison of the transcriptomes of CHIR-M-MØ versus DMSO M-MØ is shown in the bottom panel. (**E**) Relative expression of the indicated MAFB-dependent genes in CHIR-M-MØ and DMSO M-MØ (GSE185872). Mean ± SEM of 3 independent donors are shown, with indication of the *P*_adj_. Statistical significance was calculated using the R package DESeq2. (**F**) Production of soluble factors by CHIR-M-MØ and DMSO M-MØ determined by ELISA. Mean ± SEM of 3 independent donors are shown (*P < 0.05; **P < 0.01). Statistical significance was calculated using paired ratio *t* test (2 tailed). (**G**) Relative mRNA levels of specified genes (*LGMN*, *OLFML2B*, *IL10*) in M-MØ after indicated treatments, with mean ± SEM of 3 independent samples and significance (*P < 0.05; **P < 0.01) determined by 1-way ANOVA with Tukey multiple-comparison test. (**H**) Production of LGMN, CCL18, and IL10 by M-MØ after indicated treatments, as determined by ELISA, with mean ± SEM of 4 independent samples and significance (*P < 0.05; **P < 0.01; ***P < 0.005) calculated by 1-way ANOVA (Tukey multiple-comparison test).

MAFB stability and activity is controlled through GSK3β-mediated phosphorylation of their transcriptional activation domains (23, 51–55). Given the overexpression of MAFB-dependent genes in severe COVID-19 pathogenic macrophages, we next assessed the effect of the pharmacological upregulation of MAFB (using the GSK3β inhibitor CHIR99021) on the gene sets that define pathogenic macrophages in severe COVID-19. Exposure of M-MØ to CHIR99021 (CHIR99021 M-MØ; Figure 2A) led to augmented MAFB protein levels (Supplemental Figure 2A), altered expression of almost 1,000 genes (Supplemental Figure 2B), and overenrichment of M-MØ–specific genes (Supplemental Figure 2C) and MAFB-dependent genes like *CCL2*, *IL10*, *LGMN*, *CCL8*, and *SPP1* (Figure 2, B and C, and Supplemental Figure 2D). More importantly, CHIR-M-MØ exhibited a significant positive enrichment of the gene sets that define the COVID-19 lung pathogenic macrophages CD163^+^/LGMN^+^, MoAM3, and SPP1^+^ (Figure 2D) as well as enhanced production of profibrotic soluble factors like CCL2, IL-10, LGMN, CCL8, CCL18, and SPP1 (Figure 2, E and F), whose expression is markedly elevated in pathogenic lung macrophage subsets in COVID-19. Thus, pharmacological inhibition of GSK3β increases MAFB expression and reprograms macrophages toward enhanced expression of the gene signatures of macrophages associated to COVID-19 severity. Since MAFB silencing before GSK3β-inhibition in M-MØ (Supplemental Figure 2, E and F) impaired the enhanced expression of MAFB-dependent genes (Figure 2G) as well as the increased secretion of the profibrotic factors LGMN, CCL18, and IL-10 provoked by GSK3β-inhibition (Figure 2H), we could conclude that MAFB mediates the macrophage reprogramming action of GSK3β and the potentiating effect that GSK3β-inhibition has on the gene sets that characterize pathogenic macrophage subsets in severe COVID-19.

### Identification of bona fide MAFB-regulated genes in M-MØ.

**Figure 3:**
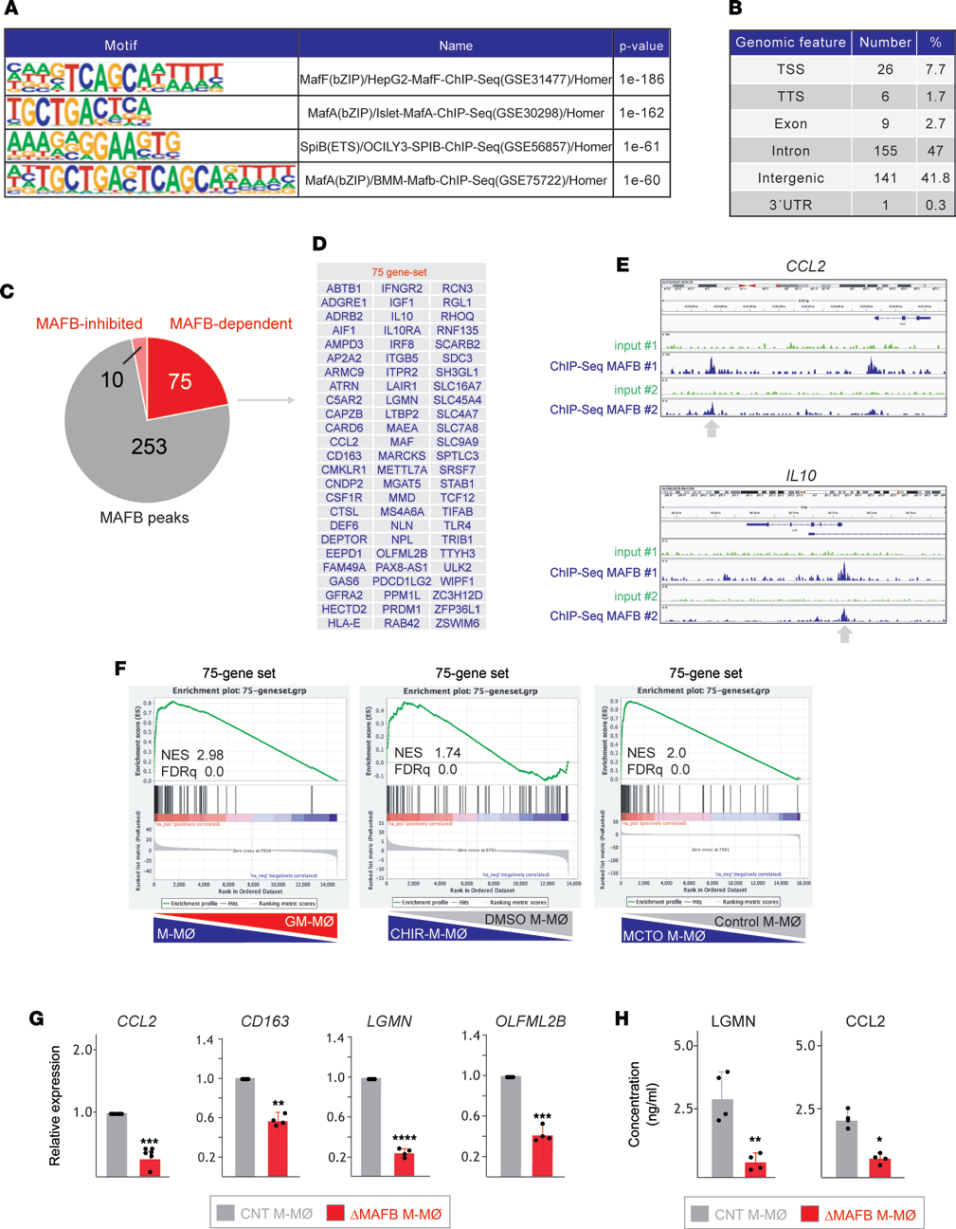
Identification of MAFB-binding elements in antiinflammatory M-MØ. (**A**) Motif enrichment within ChIP-Seq MAFB peaks, with indication of the binding sequence position weight matrices, and their corresponding statistical significance. (**B**) Summary of the location of the identified MAFB-binding sites. (**C**) Comparison of the annotated genes corresponding to ChIP-Seq peaks and MAFB-dependent and MAFB-inhibited genes. (**D**) List of the 75 genes (75-gene set) with MAFB-binding elements with expression downregulated in ΔMAFB M-MØ (MAFB-inhibited). (**E**) Viewing alignments of the MAFB-binding profiles associated with *CCL2* and *IL10* genes using the Integrative Genomics Viewer. Each track illustrates a different sample and shows the peaks obtained in 2 independent experiments with anti-MAFB antibody (ChIP-Seq MAFB #1 and MAFB #2) and the corresponding input controls (input #1, input #2). (**F**) GSEA of the 75-gene set on the ranked comparison of the transcriptomes of M-MØ versus GM-MØ (GSE68061) (left panel), CHIR-M-MØ versus DMSO M-MØ (GSE185872) (middle panel), and MCTO M-MØ versus Control MØ (GSE155883) (right panel). Normalized Enrichment Score (NES) and FDR *q* value is indicated. (**G**) Relative mRNA expression of the indicated genes in ΔMAFB M-MØ and CNT M-MØ. Mean ± SEM of 4–6 independent samples are shown (*P < 0.05; **P < 0.01; ***P < 0.001; ****P < 0.0001). Statistical significance was calculated using paired *t* test (2-tailed). (**H**) Production of LGMN and CCL2 by ΔMAFB M-MØ and CNT M-MØ, as determined by ELISA. Mean ± SEM of 4 independent samples are shown (*P < 0.05; **P < 0.01). Statistical significance was calculated using paired ratio *t* test (2-tailed).

Although MAFB-dependent genes are enriched in severe COVID-19 pathogenic macrophages, the transcriptional changes observed in ΔMAFB M-MØ or CHIR-M-MØ could result from an indirect effect of MAFB silencing/overexpression. Thus, and as a strategy to identify bona fide MAFB-dependent genes in M-MØ, we next carried out the genome-wide profiling of MAFB-binding sites in M-MØ by ChIP-Seq (Supplemental Table 4). Motif enrichment/discovery analysis of the 338 MAFB-binding sites identified in 2 independent experiments revealed a strong enrichment of MAF family–binding motifs and spleen focus forming virus proviral integration oncogene-related B–binding (SpiB-binding) elements (Figure 3A), in agreement with the reported MafB-SpiB in vitro interaction (56). In fact, the 338 MAFB-binding sites mapped to 320 annotated genes (Supplemental Table 4 and Figure 3B) and included 75 genes (termed “75-gene set”) significantly downregulated in ΔMAFB M-MØ (MAFB-dependent genes) (Figure 3, C–E) and highly enriched in M-MØ, MCTO M-MØ, and CHIR-M-MØ (Figure 3F). Indeed, the 75-gene set included genes shared by the various pathogenic monocyte-derived macrophage clusters identified in severe COVID-19 (39, 48–50), like *CCL2*, *CD163*, *CMKLR1*, *CSF1R*, *LGMN*, *MAF*, *MARCKS*, and *OLFML2B*, and whose MAFB-dependent expression was confirmed on a validation set of ΔMAFB M-MØ samples (Figure 3G) and also at the protein level (Figure 3H). Thus, we concluded that the 75-gene set includes bone fide MAFB-dependent genes whose expression reflects the expression and activity of MAFB in human macrophages.

### SARS-CoV-2 enhances the expression of MAFB and the MAFB-dependent transcriptome in human monocyte–derived macrophages.

**Figure 4:**
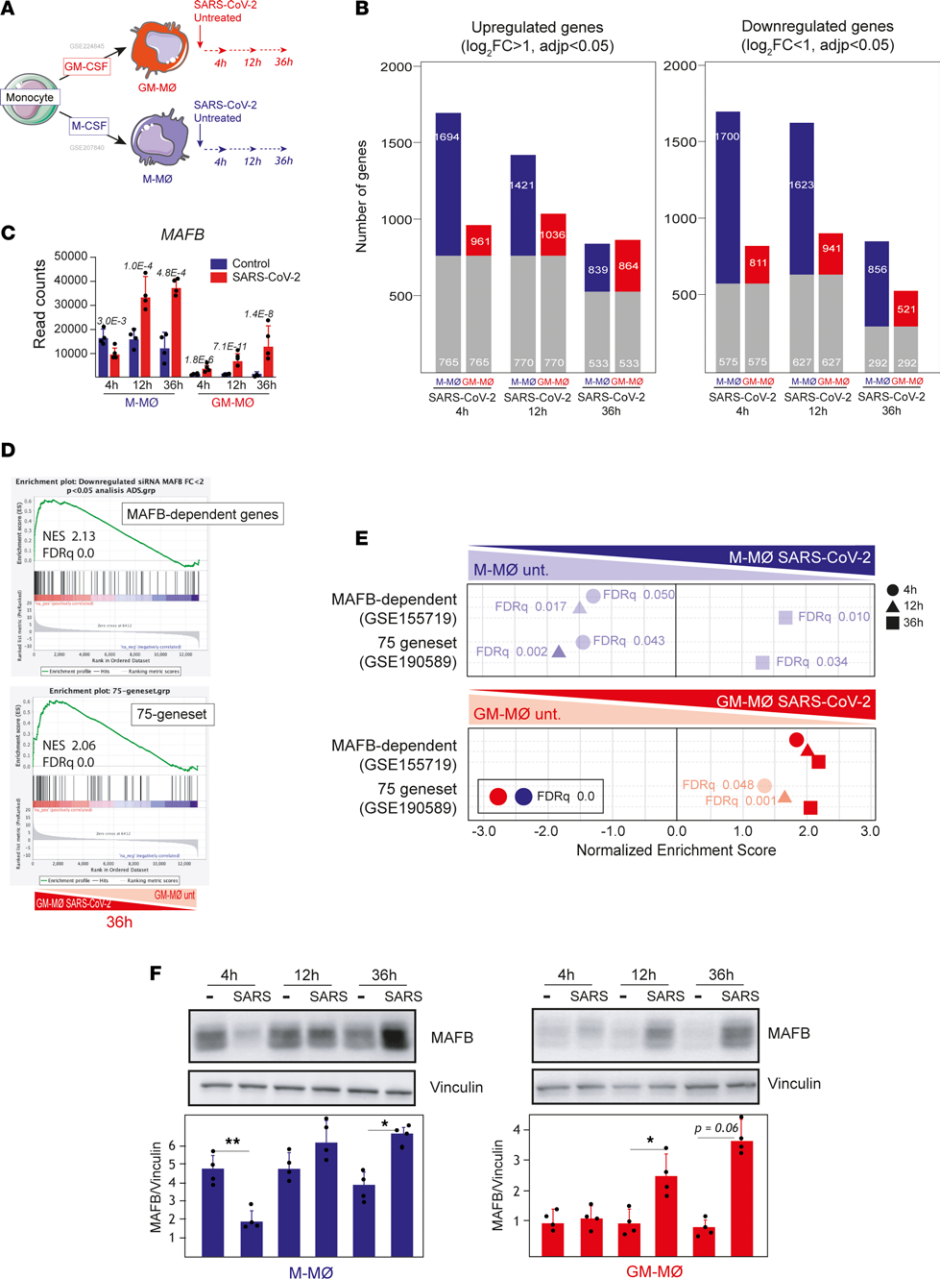
SARS-CoV-2 infection of human monocyte–derived macrophages upregulates the expression of MAFB and MAFB-dependent genes. (**A**) Schematic representation of the generation of SARS-CoV-2–infected M-MØ (M-MØ SARS-CoV-2) and GM-MØ (GM-MØ SARS-CoV-2), and their corresponding untreated controls at different times before RNA isolation and RNA-Seq (GSE207840) using 4 independent samples. (**B**) Number of differentially expressed genes ([log_2_FC] > 1; *P*_adj_ < 0.05) in SARS-CoV-2–infected macrophages (M-MØ SARS-CoV-2 and GM-MØ SARS-CoV-2) relative to uninfected controls at 4, 12, and 36 hours. Gray columns indicate the number of genes regulated in both M-MØ and GM-MØ. (**C**) *MAFB* gene expression in SARS-CoV-2–exposed or untreated M-MØ and GM-MØ at the indicated time points after viral infection and as determined in RNA-Seq experiments (GSE207840). *P*_adj_ values (relative to untreated samples) are indicated in each case. Statistical significance was calculated using the R-package DESeq2. (**D**) GSEA of MAFB-dependent genes (GSE155719) (upper panel) and the 75-gene set (GSE190589) (lower panel) on the ranked comparison of the transcriptomes of GM-MØ SARS-CoV-2 versus untreated GM-MØ, 36 hours after viral exposure. (**E**) Summary of GSEA of MAFB-dependent genes (GSE155719) and the 75-gene set (GSE190589) on the ranked comparison of the transcriptomes of M-MØ SARS-CoV-2 versus untreated M-MØ (upper panel) or GM-MØ SARS-CoV-2 versus untreated GM-MØ (lower panel), determined at 4, 12, and 36 hours after viral exposure. FDR *q* values are indicated in each case. (**F**) MAFB protein levels in M-MØ SARS-CoV-2 (left panel) and GM-MØ SARS-CoV-2 (right panel) at the indicated time points after exposure to SARS-CoV-2 (SARS) or to SARS-CoV-2 VLPs, as determined by Western blot. Vinculin protein levels were determined as protein loading control. Mean ± SEM of the MAFB/vinculin protein ratios from 4 independent experiments are shown (*P < 0.05; **P < 0.01). Statistical significance was calculated using 1-way ANOVA with Tukey multiple-comparison test. A representative Western blot experiment is shown in each case.

To assess the contribution of MAFB to human macrophage responses toward SARS-CoV-2, M-MØ and GM-MØ were exposed to SARS-CoV-2 (Wuhan strain, MOI 1), and the transcriptome of M-MØ SARS-CoV-2 and GM-MØ SARS-CoV-2 was determined at 3 different time points (4, 12, and 36 hours) (Figure 4A). Exposure to SARS-CoV-2, confirmed by the presence of viral transcripts (Supplemental Figure 3A), greatly modified the macrophage gene profile at all time points, with both macrophage types showing specific responses toward SARS-CoV-2 (Figure 4B). Importantly, M-MØ SARS-CoV-2 and GM-MØ SARS-CoV-2 significantly overexpressed the genes that characterize BALF macrophages from patients with severe COVID-19 (57, 58), as well as the gene clusters that mark monocyte-derived and alveolar macrophages from patients with COVID-19 (59) (Supplemental Figure 3B), thus emphasizing the physiological significance of these in vitro infections.

Regarding MAFB, infection of M-MØ led to diminished *MAFB* gene expression at early time points but significantly augmented *MAFB* levels 12 hours and 36 hours after SARS-CoV-2 exposure, whereas *MAFB* expression raised continuously in SARS-CoV-2–treated GM-MØ (Figure 4C). More importantly, exposure to SARS-CoV-2 significantly increased the global expression of MAFB-dependent genes and the “75-gene set,” albeit with distinct kinetics in M-MØ and GM-MØ (Figure 4, D and E). Specifically, both gene sets were downregulated by SARS-CoV-2 in M-MØ at early time points and were later enhanced at 36 hours after viral exposure, while expression of MAFB-dependent genes and the 75-gene set was significantly augmented at all time points after SARS-CoV-2 exposure of GM-MØ (Figure 4, D and E). Besides, SARS-CoV-2 infection of both M-MØ and GM-MØ enhanced expression of IL-10–dependent genes, STAT3-dependent genes, and the expression of profibrotic genes (Supplemental Figure 3, C and D). More importantly, MAFB protein expression paralleled *MAFB* gene expression levels in both macrophage subtypes upon contact with SARS-CoV-2 (Figure 4F). Therefore, exposure to SARS-CoV-2 results in enhanced expression of MAFB and MAFB-dependent genes in both M-MØ and GM-MØ at late time points. Consequently, since MAFB-dependent genes are overexpressed in pathogenic pulmonary macrophages in severe COVID-19 (50) (Figure 1), these results suggest that SARS-CoV-2–regulated MAFB is responsible for the gene expression profile that characterizes pulmonary macrophages in severe COVID-19 (either M-MØ–like monocyte-derived or GM-MØ–like lung resident macrophages).

### MAFB mediates the transcriptional and functional response of M-MØ and GM-MØ to SARS-CoV-2.

**Figure 5:**
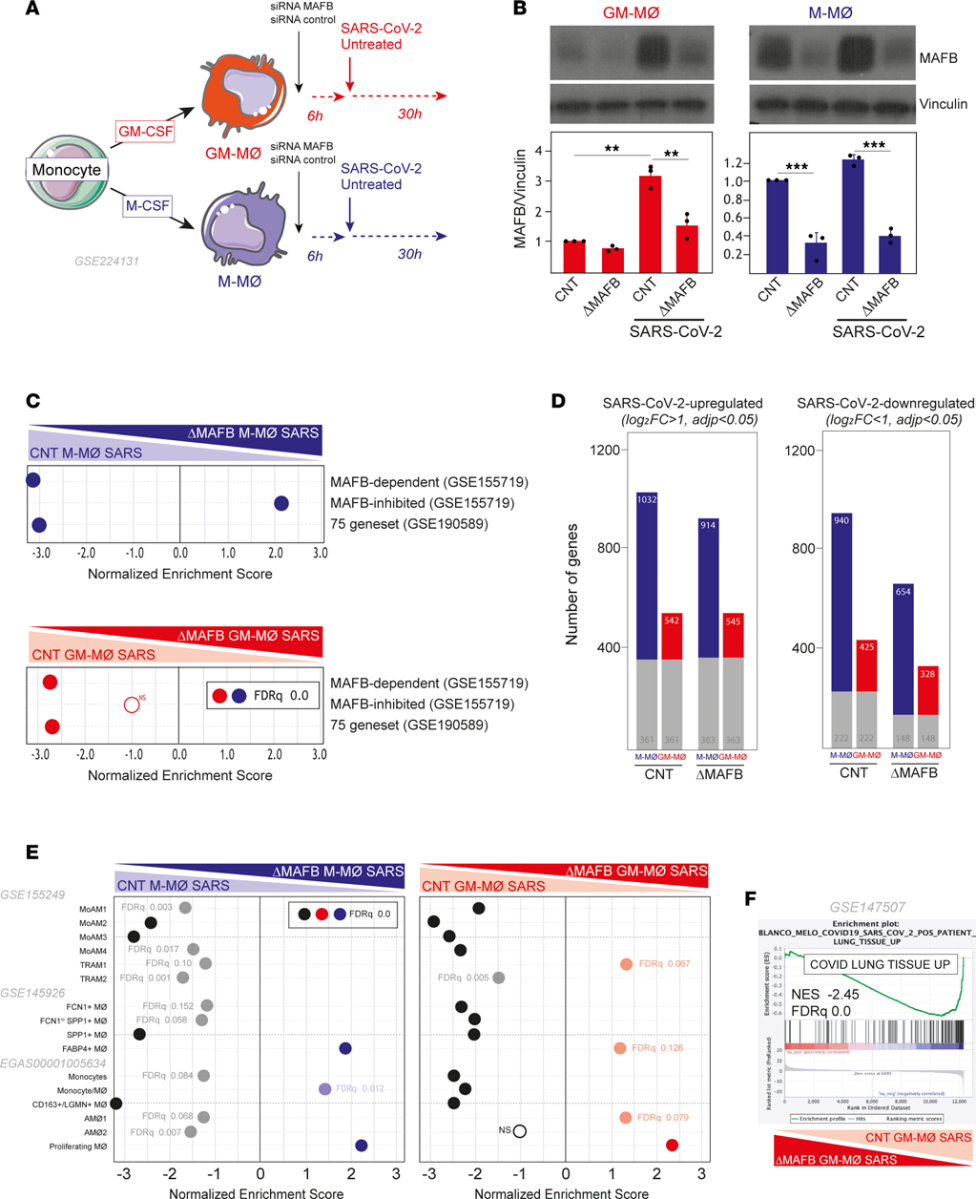
MAFB silencing drastically modifies the response of human macrophages to SARS-CoV-2. (**A**) Schematic representation of the transfection of M-MØ or GM-MØ with a MAFB-specific or control siRNA before SARS-CoV-2 exposure to generate ΔMAFB M-MØ SARS, ΔMAFB GM-MØ SARS, and their controls. (**B**) MAFB protein levels in ΔMAFB M-MØ SARS, ΔMAFB GM-MØ SARS, and their controls, as determined by Western blot, with vinculin as a loading control. Mean ± SEM of the MAFB/vinculin protein ratios from 3 independent experiments are shown (*P < 0.05; **P < 0.01; ***P < 0.001). Statistical significance was calculated using 1-way ANOVA with Tukey multiple-comparison test. A representative Western blot experiment is shown. (**C**) Summary of GSEA of MAFB-dependent genes, MAFB-inhibited genes (GSE155719), and the 75-gene set (GSE190589) on the ranked comparison of the transcriptomes of ΔMAFB M-MØ SARS and CNT M-MØ SARS (upper panel) or ΔMAFB GM-MØ SARS and CNT GM-MØ SARS (lower panel). Except where indicated, FDR *q* = 0.0 in each case. (**D**) Number of differentially expressed genes ([log_2_FC] > 1; *P*_adj_ < 0.05) in SARS-CoV-2–infected macrophages (ΔMAFB M-MØ SARS and ΔMAFB GM-MØ SARS) relative to controls (CNT M-MØ SARS and CNT GM-MØ SARS). Gray columns indicate genes regulated in both M-MØ and GM-MØ. (**E**) Summary of GSEA of the gene sets characterizing macrophage subsets identified in severe COVID-19 (39, 48, 49) on the ranked comparison of the transcriptomes of ΔMAFB M-MØ SARS and CNT M-MØ SARS (left panel) or ΔMAF GM-MØ SARS versus CNT GM-MØ SARS (right panel). (**F**) GSEA of the genes strongly upregulated (log2[FC] > 3.58; *P*_adj_<0.05) in postmortem lung tissue from patients with COVID-19 (“COVID Lung Tissue UP”; GSE147507) (60) on the ranked comparison of the transcriptomes of ΔMAFB GM-MØ SARS versus CNT GM-MØ SARS. In all panels, FDR *q* values and the source of the original gene sets are indicated.

Given the MAFB increase in infected macrophages, and as a final approach to demonstrate the involvement of MAFB in the macrophage response to SARS-CoV-2, both M-MØ and GM-MØ were exposed to SARS-CoV-2 (Wuhan strain, MOI 1) after siRNA-mediated MAFB knockdown (Figure 5A). After confirming the diminished expression of MAFB both before and 30 hours after viral infection (Figure 5B), analysis of the transcriptome of the resulting ΔMAFB M-MØ SARS and ΔMAFB GM-MØ SARS revealed that MAFB silencing not only impaired the expression of MAFB-dependent genes, CHIR99021-upregulated genes, and the 75-gene set (Figure 5C and Supplemental Figure 4A), as expected, but it drastically affected the expression of SARS-CoV-2–regulated genes in M-MØ and GM-MØ (Figure 5D). Specifically, MAFB silencing reduced the number of genes upregulated and downregulated by SARS-CoV-2 in both M-MØ and GM-MØ (Figure 5D). More importantly, GSEA showed that MAFB silencing significantly reduced the expression of the gene sets that define the profibrotic and pathogenic lung macrophage subsets SPP1^+^ MØ (Group 3, GSE145926) (49), MoAM3 (GSE155249) (39), and CD163^+^/LGMN^+^ MØ (EGAS00001005634) (48) in both SARS-CoV-2–treated M-MØ and GM-MØ (Figure 5E). Altogether, these results demonstrate that MAFB critically determines the transcriptome of SARS-CoV-2–exposed human macrophages and, particularly, the expression of genes that define profibrotic pathogenic pulmonary macrophages in severe COVID-19. Furthermore, MAFB knockdown drastically reduced the expression of the genes that are strongly upregulated (log_2_[FC] > 3.58, adjusted *P* [*P*_adj_] < 0.05) in postmortem lung tissue from patients with COVID-19 versus uninfected biopsy (60) (Figure 5F). In addition, MAFB silencing also impaired the acquisition of the genes that define the proinflammatory macrophage subsets in COVID-19 (MoAM1, MoAM2, FCN1^+^) (Figure 5E), indicating that MAFB also influences the transcriptome of the macrophage subsets that are responsible for the production of proinflammatory factors in severe COVID-19.

### MAFB contributes to the upregulated/induced expression of chemokine-encoding genes in SARS-CoV-2–exposed human macrophages.

**Figure 6:**
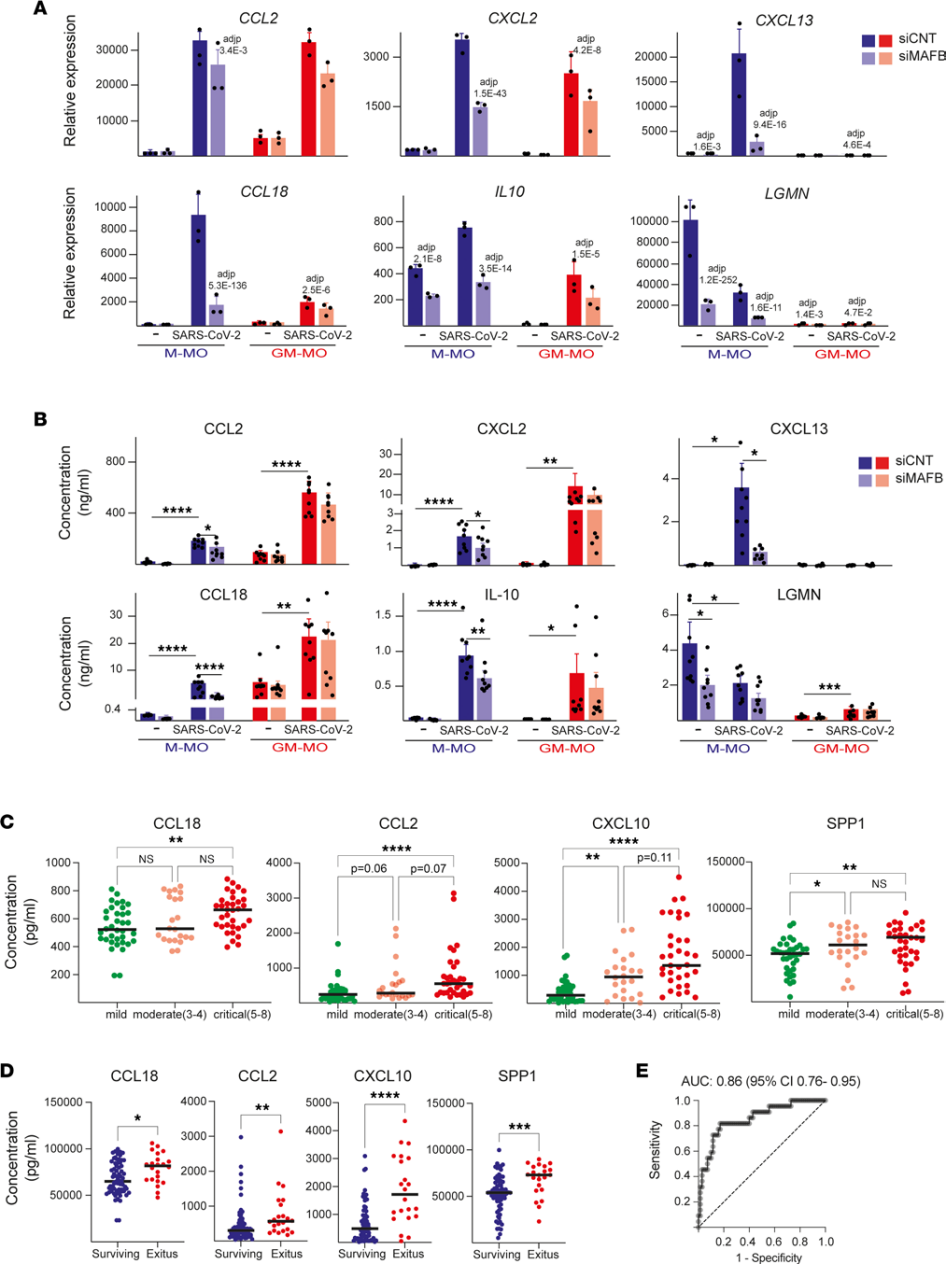
MAFB contributes to the expression of profibrotic and neutrophil-recruiting chemokines in human macrophages exposed to SARS-CoV-2. (**A**) Relative mRNA levels of the indicated genes in ΔMAFB M-MØ SARS, ΔMAFB GM-MØ SARS, and the corresponding controls, as determined by RNA-Seq. Mean ± SEM of 3 independent samples are shown. *P*_adj_ of the comparison of macrophages with or without MAFB knockdown is shown. Statistical significance was calculated using the R-package DESeq2. (**B**) Production of the indicated soluble factors in ΔMAFB M-MØ SARS, ΔMAFB GM-MØ SARS, and the corresponding controls, as determined by ELISA. Mean ± SEM of 9 independent samples are shown (*P < 0.05; **P < 0.01; ***P < 0.001, ****P < 0.0001). Statistical significance was calculated using 1-way ANOVA with Tukey multiple-comparison test. (**C** and **D**) Concentration of CCL2, CCL18, SPP1, and CXCL10 in plasma from a cohort of 92 patients with COVID-19 grouped according to their OMS classification 14 days after hospital admission (**C**) or mortality (**D**). Horizontal lines represent the medians (*P < 0.05; **P < 0.01; ***P < 0.001; ****P < 0.0001). For **C**, statistical significance (*P* values) was obtained using the Kruskal–Wallis test followed by pairwise comparisons using the Dunn’s test. For **D**, statistical significance (*P* values) was obtained using the 2-tailed Mann-Whitney *U* test. (**E**) ROC curve estimated using the plasma cytokine levels of SPP1, CCL18, and CXCL10 on hospital admission for patient survival or death during hospitalization. Death and survival predicted powers were estimated as 66.67% and 84.42%, respectively. *P* < 0.0001 for the parameters estimated. Values for AUC and its 95% CI are indicated.

The comparison of the MAFB-dependent transcriptome of M-MØ and GM-MØ before and after viral exposure showed numerous genes whose MAFB-dependency was evident in both basal conditions and after viral stimulation, including *IL10*, *CXCL12*, and *CXCL13* (Supplemental Figure 4, B and C). However, a considerable number of genes was identified whose expression was MAFB-dependent exclusively in SARS-CoV-2–exposed macrophages (ΔMAFB M-MØ SARS and/or ΔMAFB GM-MØ SARS) (Supplemental Figure 4, B and C), including genes encoding chemokines with profibrotic and monocyte-recruiting functions (e.g., *CCL3*, *CCL13*, *CCL18*) or neutrophil-attracting activity (e.g., *CXCL2*, *CXCL5*) (Figure 6A and Supplemental Figure 4D). Therefore, MAFB also regulates the expression of pathologically significant chemokines in SARS-CoV-2–exposed human macrophages. Indeed, MAFB knock-down was sufficient to impair the virus-stimulated production of IL-10 and the chemokines CXCL2, CXCL13, and CCL18 (Figure 6B), thus emphasizing that MAFB controls the expression of both profibrotic factors (IL-10, CCL18, CXCL13) and neutrophil-attracting chemokines (CXCL2) in human macrophages exposed to SARS-CoV-2. These findings are particularly remarkable because a strong chemokine expression has been consistently observed in in vitro, ex vivo, and in vivo models of SARS-CoV-2 infection (60), because some of these chemokines are biomarkers for COVID-19 severity (61–71), and because fibrosis is a pathogenic parameter in severe COVID-19 (48), where neutrophilia contribute to pathological complications (72, 73). In addition, MAFB knockdown impaired the expression of *SPP1* in M-MØ and in SARS-CoV-2–treated GM-MØ (Supplemental Figure 4D), which is particularly relevant because *SPP1* marks pathogenic macrophages in COVID-19 (49) and SPP1 plasma levels are high in severe COVID-19 and predict the need for ICU transfer (74).

Finally, since MAFB-dependent factors like IL-10, SPP1, CCL2, and CXCL13 are biomarkers for COVID-19 severity (63, 65–71, 74, 75), we next assessed whether additional MAFB-dependent soluble factors might also predict COVID-19 severity or outcome. To that end, and after analysis of an exploratory cohort of 58 patients with COVID-19 (data not shown), the plasma levels of soluble factors encoded by MAFB-dependent genes were determined in plasma from a cohort of 92 patients with COVID-19 differing in their OMS classification. Like SPP1, CXCL10, and CCL2, whose plasma levels associate with COVID-19 severity (63, 65–71, 74, 75), the plasma level of CCL18 was also found to be significantly different between patients with mild and critical COVID-19 (Figure 6C). Moreover, plasma classification according to the patient outcome revealed that the plasma levels of SPP1, CCL18, CCL2, and CXCL10 were also significantly different between patients who died and those who survived (Figure 6D). We further analyzed, by logistic regression, whether these cytokines could be used as prognostic predictors of COVID-19 mortality. The ROC curve of each single cytokine was calculated using the expression levels upon hospital admission. Results show that the AUC for the 4 assessed cytokines varied from 0.6721 (for CCL18) to 0.7955 (for CXCL10) (data not shown). We next tested different combinations of the 4 cytokines for the prediction of disease death and found that the combination of SPP1, CCL18m and CXCL10 best discriminated between survival and death of patients with COVID-19 (AUC of 0.86) (Figure 6E). This result indicates that the combined use of SPP1, CCL18, and CXCL10 provides a powerful immune predictor signature of COVID-19 mortality. Therefore, MAFB controls the expression of soluble factors that significantly contribute to COVID-19 pathogenesis (monocyte recruitment, fibrosis) and that constitute good predictors for COVID-19 severity and outcome.

## Discussion

Transcriptional analysis of the dominant macrophage clusters in lungs from patients with COVID-19 led us previously to hypothesize that MAFB shapes the gene profile of the pulmonary macrophages that drive severe COVID-19 pathogenesis (50), a hypothesis later supported by the transcriptome of monocytes exposed to SARS-CoV-2 (48). Following the identification of a set of MAFB-regulated genes (75-gene set) in monocyte-derived macrophages by ChIP-Seq, we have now directly examined the involvement of MAFB in the response of human macrophages to SARS-CoV-2 infection by means of genetic and pharmacological approaches. Our results indicate that MAFB exhibits a dual role in macrophages, as it is required for the maintenance of the antiinflammatory functions of nonstimulated monocyte-derived macrophages but also contributes to the acquisition of a full profibrotic and proinflammatory profile in SARS-CoV-2–exposed macrophages. Indeed, knock-down of MAFB prior to SARS-CoV-2 exposure significantly reduces the expression of chemokines that stimulate fibrosis (CXCL13, CCL18) and neutrophil recruitment (various CXCL chemokines), 2 processes that are closely linked to COVID-19 severity and post–COVID-19 pulmonary sequelae (76). Likewise, MAFB is necessary for optimal expression of soluble factors that predict COVID-19 severity and outcome, including CCL18 as well as CCL2, CXCL10, CXCL13, and SPP1 (63, 65–71, 74, 75). As a whole, our findings demonstrate that MAFB significantly contributes to the acquisition of the gene profile and effector functions (cytokine/chemokine production) of the pathogenic macrophage subsets that promote pulmonary inflammation and fibrosis in severe COVID-19. Since single-cell transcriptomics on lungs from patients with long COVID with fibrosis has revealed a decrease in lung-resident alveolar macrophages and an increase in monocyte-derived macrophages with enhanced expression of various MAFB-dependent genes (CCL2, CCL8, CCL18, STAB1) (77), our results on the MAFB-dependent macrophage transcriptome might be also applicable to the case of lung pathogenic macrophages in long COVID.

The main complication of COVID-19 is the continuation of severe pulmonary sequelae after SARS-CoV-2 infection that includes fulminant lung fibrosis (78) and post–COVID-19 pulmonary fibrosis (PCPF) (79). These clinical entities share pathological and immune features with idiopathic pulmonary fibrosis (IPF), a paradigmatic chronic progressive fibrosing disease whose chemokine biomarkers include CCL18 and CXCL13 (80–84). Importantly, IPF and the pulmonary complications in COVID also share the presence of similar pathogenic pulmonary macrophage subsets, most of which are monocyte derived (49). As hypothesized, our findings indicate that MAFB knockdown in nonstimulated M-MØ leads to diminished expression of the markers that best define the pathogenic profibrotic macrophage subsets in severe COVID-19 (CCL2, LGMN, CD163, SPP1) (39, 48, 49), whose encoding genes contain functional MAFB-binding sites, and it leads to reduced expression of genes coding for various chemokines and other COVID-19 severity biomarkers (e.g., *CCL2*, *CXCL10*, *SPP1*, *CCL4*, *CCL5*, *CCL7*, *CD16*, *CXCL1*, *CXCL3*, *CXCL8*, *CXCL12*, *HAVCR2*, *IL2RA*, *IL10*, *IL18*) (61, 64, 85–96). In line with these effects, gene ontology analysis (ClusterProfiler; ref. 97) of the 75-gene set identified by MAFB ChIP-Seq in nonstimulated M-MØ yielded a significant enrichment of terms related to regulation of leukocyte chemotaxis and migration (data not shown).

Unexpectedly, we have also observed that MAFB controls the expression of genes regulated (up or down) upon SARS-CoV-2 exposure in both M-MØ and GM-MØ — including the expression of CCL18 and CXCL13, biomarkers for IPF (80–84, 98, 99) — and genes with expression that now appears as a predictor for COVID-19 severity and outcome. The capacity of MAFB to affect the expression of a distinct range of genes in nonstimulated and virus-stimulated macrophages might derive from the inhibitory effect that MAFB has on the expression of type I IFN and on the generation of antiviral responses (100, 101). The distinct transcriptional role of MAFB in nonstimulated and virus-stimulated macrophages might be due to its promiscuous dimerizing ability (23). Like other members of the large-MAF subfamily, MAFB can heterodimerize with members of the AP-1 superfamily of transcription factors (23), at least in vitro. Specifically, MAFB can dimerize with JUN, FOS, and FRA1/2 (23). Therefore, it is conceivable that MAFB shifts the transcriptional functions of AP-1 factors by altering the availability of factors like JUN or FOS, which are major effectors of MAPKs during macrophage activation (102, 103). If this is true, the influence of MAFB on the expression of genes encoding inflammatory chemokines in SARS-CoV-2–exposed macrophages would be explained by its ability to interact with AP-1 family partners, which largely determine the macrophage inflammatory outcome elicited by PAMP receptors (102, 103) and are major effectors of MAPKs, whose activity governs the occurrence of the “cytokine storm” during viral responses (104–106).

The infection by SARS-CoV-2 is not only dependent on the macrophage polarization state but also modulates the macrophage inflammatory potential. Thus, macrophage uptake of SARS-CoV-2 viral RNA by efferocytosis prevents their antiinflammatory repolarizaton, which enhances their inflammatory potential (107). On the other hand, the macrophage polarization state might be relevant for the outcome of SARS-CoV-2 infection. Although some studies have found that human macrophage polarization is not critical for SARS-CoV-2 infection in vitro (108), analysis of mouse alveolar macrophages and human THP-1 cells indicate that SARS-CoV-2 mostly replicates in LPS- and IFN-γ–treated (M1-like) macrophages but not in IL-4–polarized (M2-like) macrophages and might be responsible for early viral control and limiting SARS-CoV-2 spread (109, 110). However, viral RNA, by inducing the release of proinflammatory cytokines, may favor macrophage polarization toward an M1-like phenotype. Consequently, if viral load reaches a certain level in alveoli, SARS-CoV-2 might reprogram macrophages toward the M1 phenotype, thus facilitating viral spread (110). Along the same line, M1-like macrophages generated from pluripotent stem cells are more potent producers of inflammatory factors than their corresponding M2-like counterparts (111). Based on the levels of SARS-CoV-2 RNA fragments detected in RNA-Seq experiments, our results suggest that, compared with M-CSF–conditioned monocyte-derived macrophages, GM-CSF–conditioned macrophages are either less permissive for viral entry, which is in agreement with their lower level of various SARS-CoV-2 attachment factors, or are more efficient in removing intracellular viral RNA

Macrophage reprogramming, physiologically required for tissue injury removal and return to homeostasis, has also been proposed as a therapeutic target for inflammatory disorders (112). Consequently, the identification of the factors that govern macrophage specialization is a requirement before macrophage-centered therapies for inflammatory and infectious diseases can be implemented. We have found that the maintenance of the antiinflammatory profile of nonstimulated macrophages and the acquisition of profibrotic/proinflammatory functions of virus-stimulated macrophages are MAFB dependent. Consequently, MAFB constitutes a target for macrophage reprogramming. In this regard, since GSK3β inhibition potentiates the profibrotic phenotype in monocyte-derived macrophage through MAFB, the pharmacological modulation of the GSK3β/MAFB axis appears as a promising strategy for macrophage reprogramming in COVID-19. The presence of the M-CSF receptor-encoding gene *CSF1R* within the 75-gene set is particularly relevant because M-CSF is required for tissue-resident and monocyte-derived macrophage differentiation (8, 9, 113–116) and because M-CSF prompts the generation of macrophages with an antiinflammatory, trophic, immunosuppressive, and profibrotic profile (9, 13, 14, 117–125). Therefore, the link between MAFB and *CSF1R* expression further supports the notion of MAFB as a target for macrophage reprogramming.

In summary, the identification of MAFB-dependent genes and functions in human monocyte–derived macrophages, which become the major pulmonary macrophage population during COVID-19, has shown that MAFB shapes the macrophage transcriptome under both basal and virus-stimulated conditions; it also demonstrates that MAFB mediates the acquisition of the proinflammatory and profibrotic profile of pathogenic macrophages in severe COVID-19 and regulates the production of chemokines implicated in neutrophil recruitment, a driving factor for post-COVID-19 interstitial lung disease (76).

### Limitations of the study.

While we have done extensive comparison with macrophage subsets identified in BALF and pulmonary macrophages from severe COVID-19, we acknowledge that our transcriptional and functional studies have been solely performed on in vitro–generated monocyte-derived macrophages and have not analyzed lung-derived primary macrophages (either resident or recruited). This fact does not reduce the significance and relevance of our results (that is, the involvement of MAFB in macrophage responses toward SARS-CoV-2), whose generation on ex vivo macrophages would have severe logistical and ethical constraints. Besides, while in vitro–generated macrophages do not capture the whole complexity and variability inherent in the in vivo environment, they have been instrumental in identifying the molecular mechanisms underlying macrophage dysfunction in diverse pathological settings.

## Methods

### Generation of human monocyte–derived macrophages in vitro and treatments.

Human peripheral blood mononuclear cells (PBMCs) were isolated from buffy coats from anonymous healthy donors over a Lymphoprep (Nycomed Pharma) gradient according to standard procedures. Monocytes were purified from PBMC by magnetic cell sorting using anti-CD14 microbeads (Miltenyi Biotec). Monocytes (>95% CD14^+^ cells) were cultured at 0.5 × 10^6^ cells/mL in RPMI 1640 (Thermo Fisher Scientific) medium supplemented with 10% FBS (Biowest) (complete medium) for 7 days in the presence of 1,000 U/mL GM-CSF or 10 ng/mL M-CSF (ImmunoTools) to generate GM-MØ or M-MØ, respectively (20). Cytokines were added every 2 days, and cells were maintained at 37°C in a humidified atmosphere with 5% CO_2_ and 21% O_2_. SARS-CoV-2 infection was performed in the biosafety level 3 (BSL-3) facility at Imas12, using the SARS-CoV-2 clinical isolate Gisaid EPI_ISL_1120962, corresponding to ancestral S D614G. The viral stock was produced in a monolayer of Vero cells, maintained in DMEM at 37°C in a 5% CO_2_ atmosphere. Viruses were concentrated using Amicon Ultra Centrifugal Filters (100 kDa MWCO, Merck) by centrifugation at 4,000*g* for 30 minutes at 4°C (48). Final titer was estimated by virus focus forming assay on Vero E6 cells (126). In total. 1 × 10^6^ monocyte-derived macrophages in 24-well plates, kept in complete culture medium, were exposed to SARS-CoV-2 virus at MOI of 1, and cells were maintained for 4, 12, or 36 hours without medium replacement. When indicated, M-MØ were exposed to the GSK3β inhibitor CHIR99021 (10 μM) or DMSO as control. Human cytokine production was measured in M-MØ culture supernatants using commercial ELISA (CCL2 [BD Biosciences] and IL-10, CCL8, CCL18, CXCL2, CXCL5, CXCL13, LGMN and SPP1 [R&D Systems]) and following the procedures supplied by the manufacturers.

### Quantitative PCR.

Total RNA was extracted using the total RNA and protein isolation kit (Macherey-Nagel). RNA samples were reverse transcribed with High-Capacity cDNA Reverse Transcription reagents kit (Applied Biosystems) according to the manufacturer’s protocol. Quantitative PCR (qPCR) was performed with LightCycler 480 Probes Master (Roche Life Sciences) and TaqMan probes on a standard plate in a Light Cycler 480 instrument (Roche Diagnostics). Gene-specific oligonucleotides (Supplemental Table 1) were designed using the Universal ProbeLibrary software (Roche Life Sciences). Results were normalized to the expression level of the endogenous references genes *TBP* and *HPRT1* and were quantified using the ΔΔCT method.

### Western blot.

Cell lysates were subjected to SDS-PAGE (50 μg unless indicated otherwise) and transferred onto an Immobilon-P polyvinylidene difluoride membrane (PVDF; MilliporeSigma). After blocking the unoccupied sites with 5% nonfat milk diluted in Tris-Buffered Saline plus Tween 20 (TBS-T), protein detection was carried out with antibodies against MAFB (HPA005653, Sigma-Aldrich) or vinculin (V9131, Sigma-Aldrich) as a protein loading control. Quimioluminiscence was detected in a Chemidoc Imaging system (Bio-Rad) using SuperSignal West Femto (Thermo Fisher Scientific).

### siRNA transfection.

M-MØ (1 × 10^6^ cells) were transfected with a human *MAFB*-specific siRNA (siMAFB, 25 nM) (Dharmacon) or a human *MAF*-specific siRNA (siMAF, 25 nM) (Dharmacon) using HiPerFect (Qiagen). Silencer Select Negative Control No. 1 siRNA (siCtrl, 25 nM) (Dharmacon) was used as negative control siRNA. Six hours after transfection, cells were either allowed to recover from transfection in complete medium (18 hours), or exposed to SARS-CoV-2 for 30 additional hours, and lysed. Knockdown of MAFB was confirmed by qPCR and Western blot.

### RNA-Seq and data analysis.

RNA was isolated from M-MØ transfected with either *MAF*-specific siRNA (ΔMAF M-MØ), *MAFB*-specific siRNA (ΔMAFB M-MØ), or control siRNA (CNT M-MØ), as well as from M-MØ generated from monocytes from a patient with multicentric carpotarsal osteolysis (MCTO, MCTO M-MØ) or healthy controls, and subjected to sequencing on a BGISEQ-500 platform (http://www.bgitechsolutions.com/). Additionally, RNA from M-MØ exposed to the GSK3β inhibitor CHIR99021 (10 μM) or DMSO was isolated and similarly processed for sequencing on a BGISEQ-500 platform. Following the same procedure, RNA-Seq was performed on M-MØ or GM-MØ cultured with or without SARS-CoV-2 for 4, 12, and 36 hours — or on SARS-CoV-2–treated ΔMAFB M-MØ, ΔMAFB GM-MØ, and CNT M-MØ — using the BGISEQ-500 platform. RNA-Seq data were deposited in the Gene Expression Omnibus (http://www.ncbi.nlm.nih.gov/geo/) under accession no. GSE155719 (siRNA-transfected M-MØ), GSE155883 (MCTO M-MØ), GSE185872 (CHIR99021-treated M-MØ), GSE207840 (SARS-CoV-2–infected M-MØ), GSE224845 (SARS-CoV-2–infected GM-MØ), and GSE224131 (SARS-CoV-2–infected ΔMAFB macrophages). Low-quality reads and reads with adaptors or unknown bases were filtered to get the clean reads. Sequences were mapped to GRCh38 genome using HISAT2 (127) or Bowtie2 (128), and clean reads for each gene were calculated using htseq-count (129) and the RSEM software package (130). SARS-CoV-2 fragments were mapped to the SARS-CoV-2 NCBI reference genome NC_045512.2 and quantified by using the Subread software package (131). Differential gene expression was assessed by using the R-package DESeq2 (pairing donors for the siMAF and siMAFB experiments). Differentially expressed genes were analyzed for annotated gene sets enrichment using ENRICHR (http://amp.pharm.mssm.edu/Enrichr/) (132, 133), and enrichment terms considered significant with a Benjamini-Hochberg *P*_adj_ < 0.05. For gene set enrichment analysis (GSEA) (http://software.broadinstitute.org/gsea/index.jsp) (134), gene sets available at the website, as well as gene sets generated from publicly available transcriptional studies (https://www.ncbi.nlm.nih.gov/gds), were used. The gene sets that define the transcriptome of human monocyte–derived proinflammatory GM-MØ (“Proinflammatory gene set”) or antiinflammatory M-MØ (“Antiinflammatory gene set”) have been previously reported (GSE68061) (12, 14). The data sets used throughout the manuscript (either reported here or previously published by our group or others) are listed and described in Supplemental Table 2.

### ChIP-Seq and ChIP-Seq bioinformatic analysis.

ChIP-Seq was performed essentially as described by Nowak et al. (135), using a Diagenode Bioruptor for sonication and using DNA crosslinking using 1% formaldehyde and the rabbit anti-MAFB antibody (HPA005653, MilliporeSigma) for immunoprecipitation. Sequencing of ChIP-Seq–derived libraries was performed on the BGI-500 platform. Sequenced single-end 50 bp reads were aligned to the genome assembly GRCh38 using BWA program (136). Homer software suite was used for peak calling, peak annotation, and motif discovery (137) (http://homer.ucsd.edu/homer/). For peak calling analysis, “blacklist” peaks were filtered out according to ref. 138. The Integrative Genomics Viewer (IGV) genome browser was used to visualize the aligned read files and the identified peaks (139) (https://software.broadinstitute.org/software/igv/). ChIP-Seq data have been deposited in GEO under accession no. GSE190589.

### Demographic and clinical characteristics of the study population and sample collection.

A total of 92 patients with COVID-19, who attended the emergency department of the University Hospital La Princesa during October 2020-January 2021, were included in the study. The main outcome was the World Health Organization (WHO) COVID-19 severity scale (140), at 14 day follow-up after admission, grouped in Mild (levels 1 to 3), Moderate (level 4) and Severe (levels 5 to 8), as previously described (141). The median age was 70 years (IQR= 55-79.75), 57.80% were males and 86.96% were Caucasian (see Supplemental Table 3). Plasma samples were collected at hospital admission, obtained by sedimentation, heated at 56ºC for 20 minutes, frozen at –20°C and stored in the Biobank facilities of the University Hospital La Princesa (ISCIII B.0000763).

### Statistics.

Statistical analyses were conducted using the GraphPad Prism software. For comparison of means, and unless otherwise indicated, statistical significance of the generated data was evaluated using a 1-way ANOVA with Tukey multiple-comparison test, paired Student *t* test (2-tailed) or paired ratio *t* test (2-tailed). In all cases, *P* < 0.05 was considered statistically significant. Intergroup clinical data comparisons for continuous variables were performed using the 2-tailed Mann-Whitney *U* test for 2 groups or using the Kruskal–Wallis test for 3 groups followed by pairwise comparisons using the Dunn’s test. Univariable and multivariable logistic regression models were used to explore the association between the expression levels of potential clinical COVID-19–relevant biomarkers with patient survival/death. The predictive values of the models were assessed by receiver operating characteristic (ROC) analysis performed with calculations of the area under the ROC curve (AUC).

### Study approval.

Samples and data from patients included in this study were provided by the Biobank University Hospital La Princesa (ISCIII B.0000763). They were processed following standard operating procedures with the appropriate approval of the Ethics and Scientific Committees (register no. 4267) and following the ethical principles established in the Declaration of Helsinki. Due to the COVID-19 pandemic and as proposed by the Spanish Agency for Medicines and Medical Devices (AEMPS), all included patients (or their representatives) only gave oral consent for their deidentified data to be used for scientific research (The Spanish Agency for Medicine and Health Products [Agencia Española de Medicamentos y Productos Sanitarios, AEMPS]; ref. 142), and the consent was registered in the electronic clinical chart.

### Data availability.

The data set supporting the conclusions of this article is available in the Gene Expression Omnibus repository (http://www.ncbi.nlm.nih.gov/geo/) under accession nos. GSE155719, GSE185872, GSE190589, GSE207840, GSE224845, and GSE224131. The Supporting Data Values file contains all data points shown in graphs.

## Author contributions

MSF, IR, CH, FL, NL, JL, and ADS performed research and analyzed data; RD, MC, APK, ADS, MAV, and ALC designed research and analyzed data; MB, SFDCO, PDW, ERV, and EFR collected patient samples and clinical data; and ADS, MAV, and ALC wrote the paper.

## Supplementary Material

Supplemental data

Supplemental tables 1-4

Supporting data values

## Version 1 11/02/2023

In-Press Preview

## Version 2 12/22/2023

Electronic publication
