# Supplementary material for: MAFB shapes human monocyte–derived macrophage response to SARS-CoV-2 and controls severe COVID-19 biomarker expression
Source: JCI Insight. 2023 Dec 22;8(24):e172862. doi: 10.1172/jci.insight.172862 (PMC10807725; doi:10.1172/jci.insight.172862)
Supplement: Supplemental data [file jciinsight-8-172862-s080.pdf]

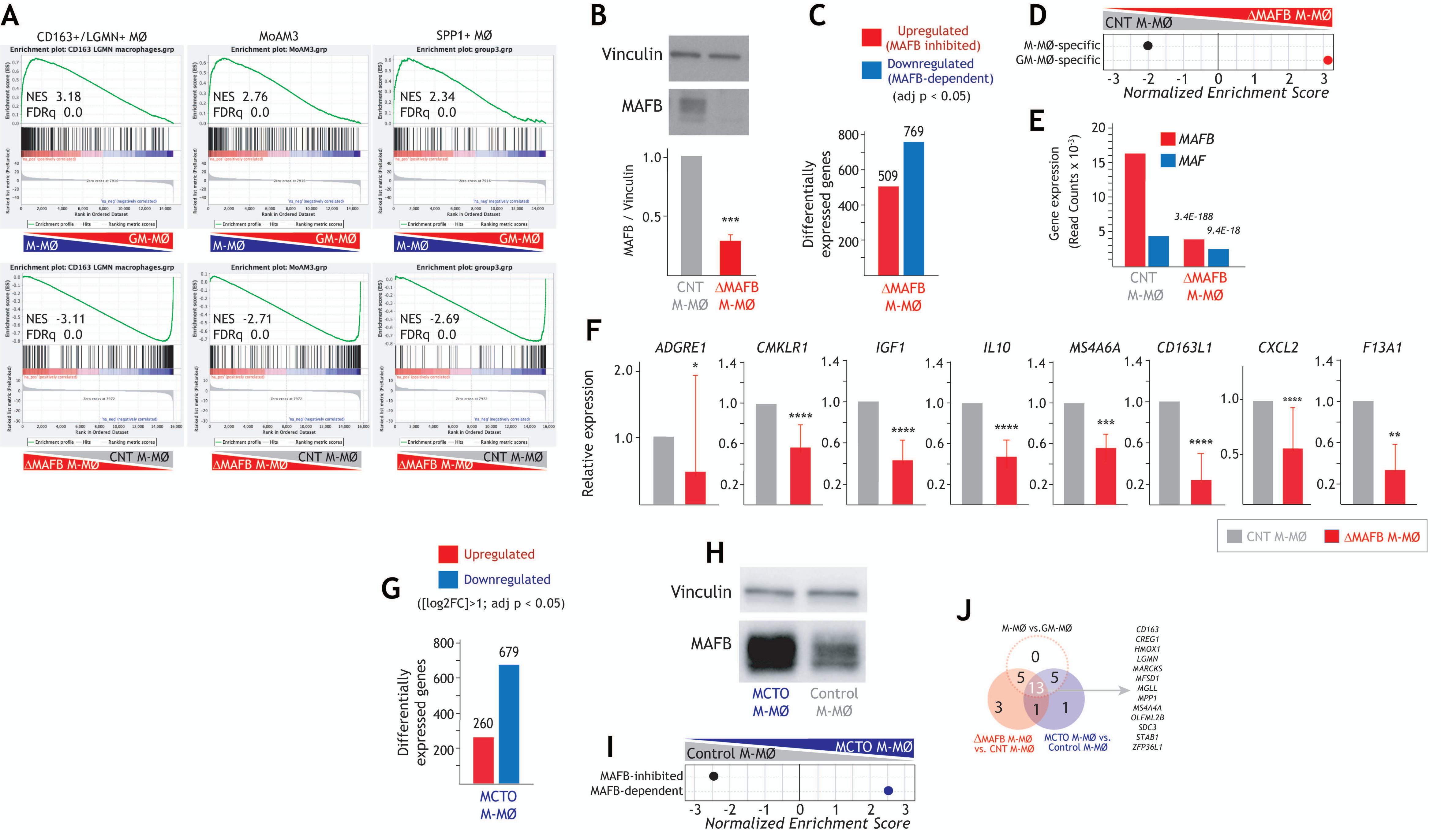

Supplementary Figure 1

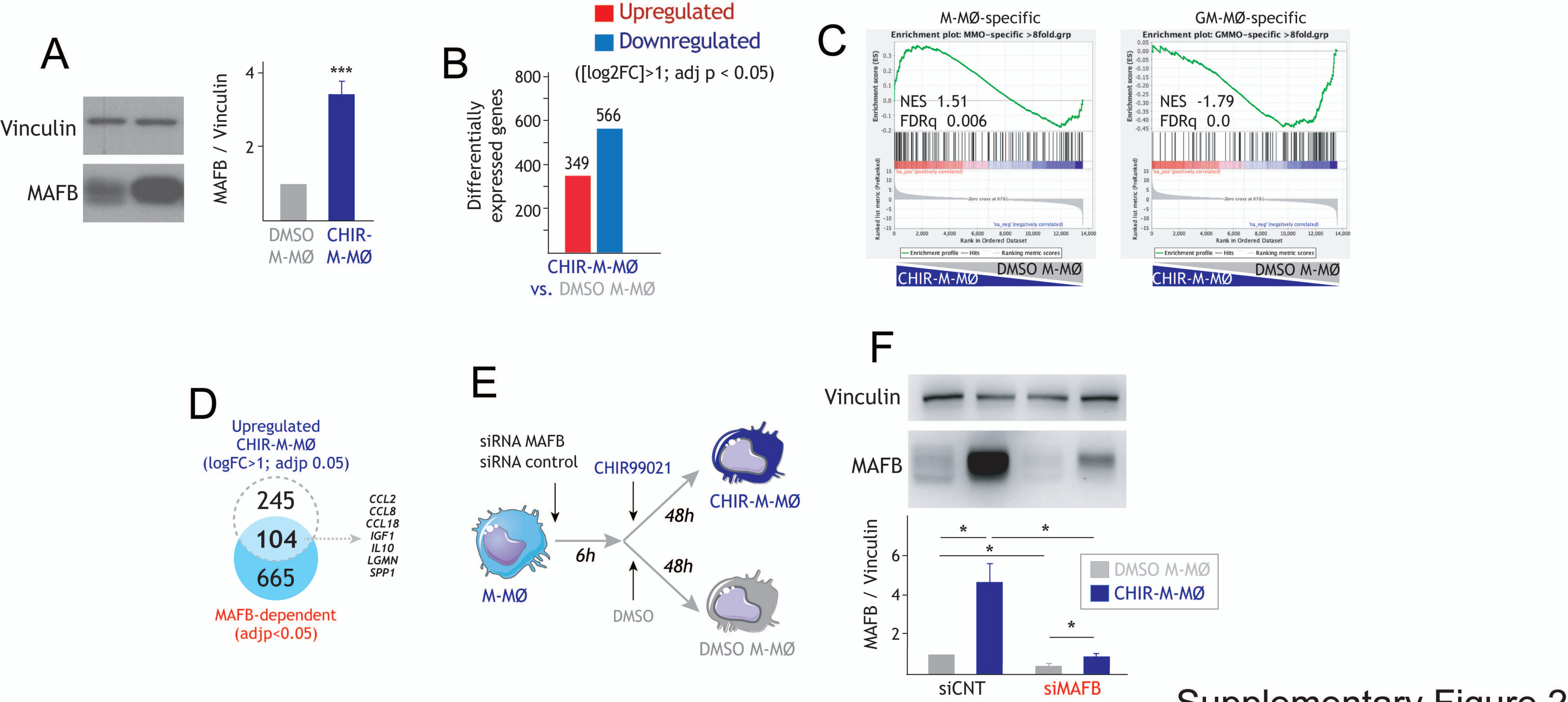

Supplementary Figure 2

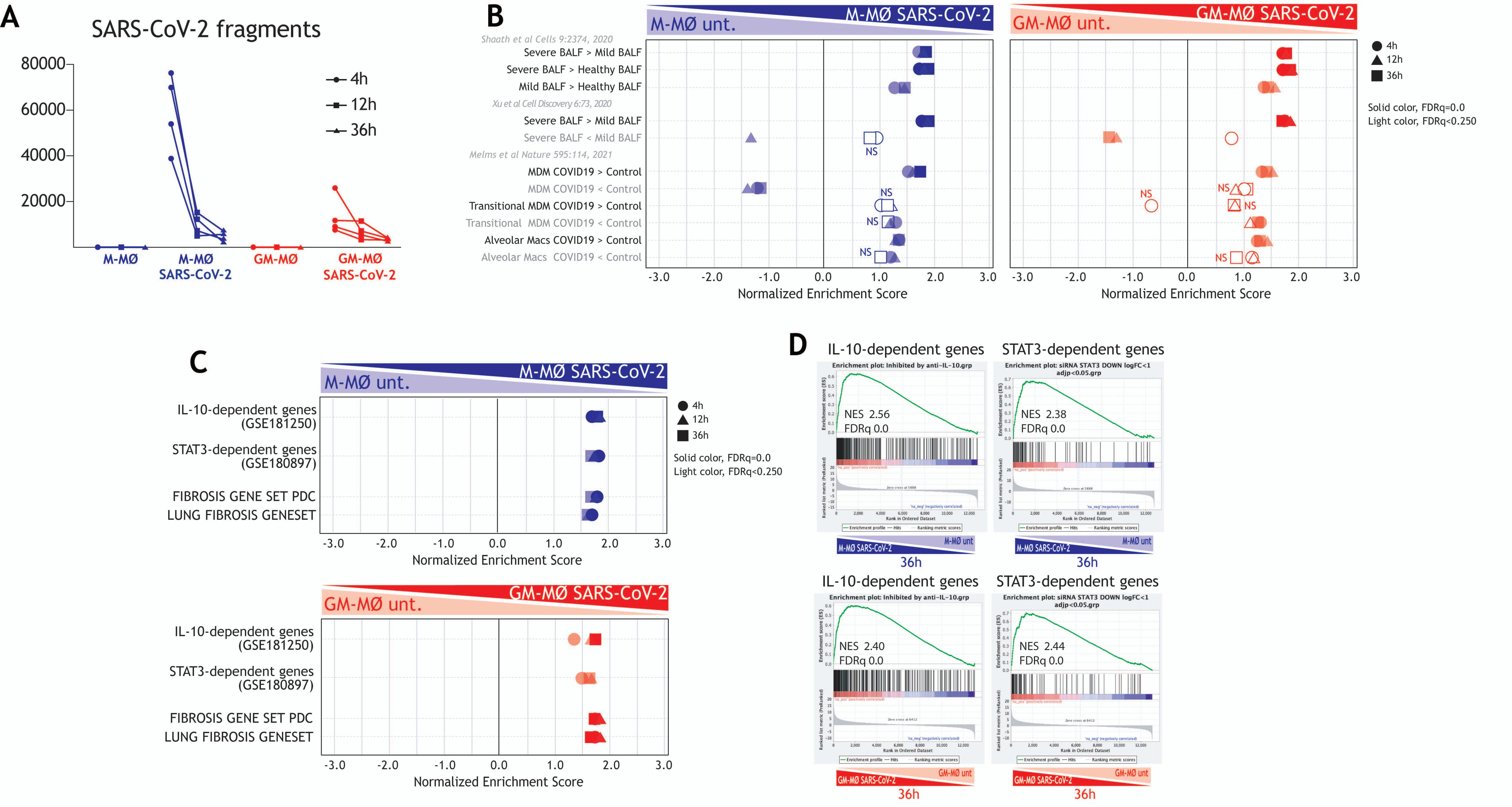

Supplementary Figure 3

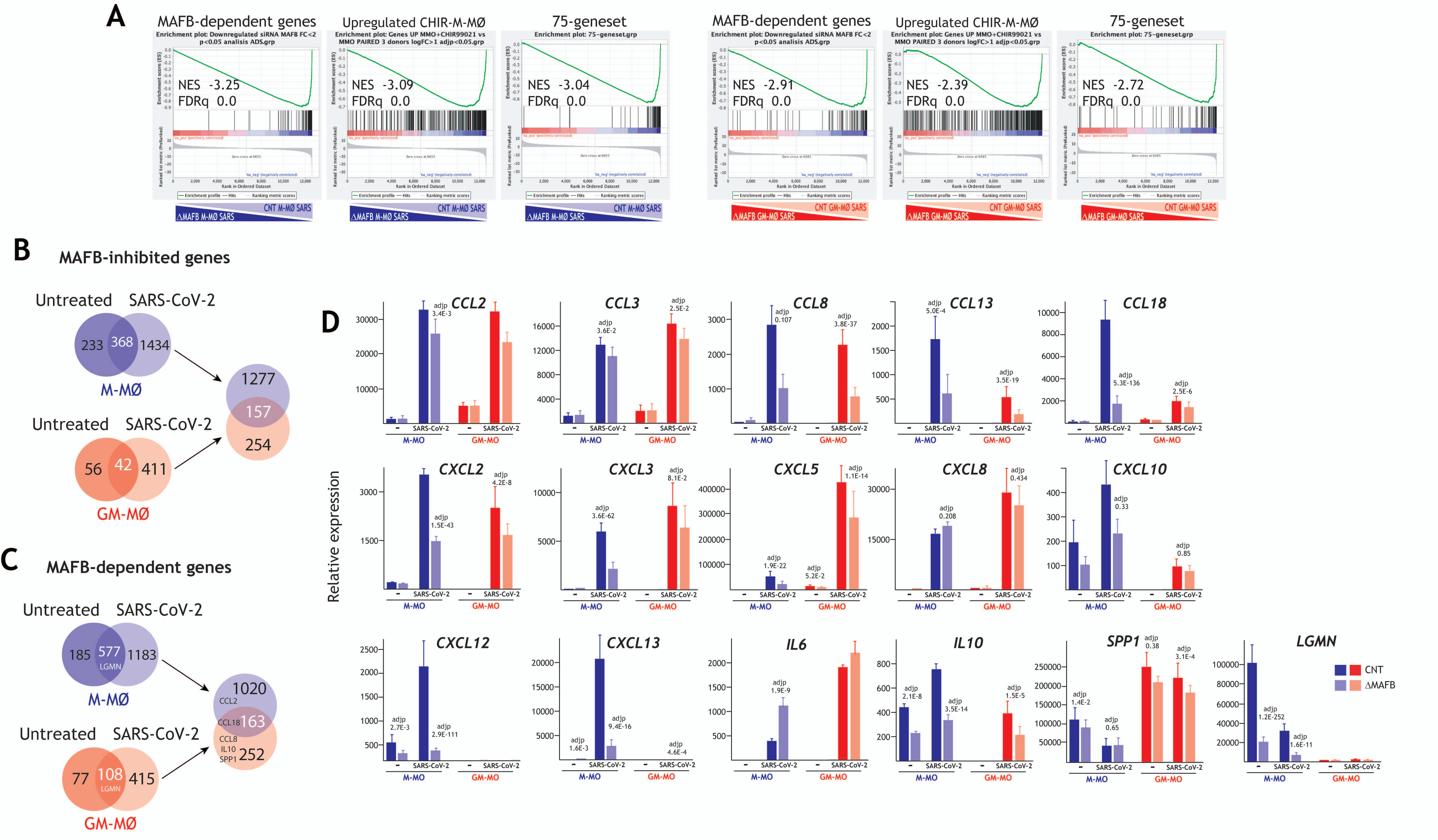

Supplementary Figure 4
